# Partner support and relationship quality as potential resources for childbirth and the transition to parenthood

**DOI:** 10.1186/s12884-023-05748-6

**Published:** 2023-06-13

**Authors:** Lisa Hoffmann, Norbert Hilger, Elena Riolino, Annika Lenz, Rainer Banse

**Affiliations:** Department of Psychology, University of Bonn, Kaiser-Karl-Ring 9, 53111 Bonn, Germany

**Keywords:** Romantic relationship, Birth, Birth experience, Transition to parenthood, COVID-19

## Abstract

**Background:**

The aim of the present paper was to explore the role of partners for the stressful life events of birth and the transition to parenthood.

**Methods:**

In a first prospective longitudinal study (*N* = 304 dyads) we tested whether relationship quality positively predicted fewer interventions during labor and birth, a more positive birth experience, and better well-being during the first six weeks after birth. In a second study we surveyed mothers (*N* = 980; retrospective quasi-experimental design) who had given birth during the first lockdown of the COVID-19 pandemic in spring 2020 – some in the absence of their partners – to test the assumption that regardless of relationship quality, the presence of the partner was positively related to low-intervention births and the birth experience.

**Results:**

The results of the longitudinal study (Study 1) could be integrated into a Single Indicator model. They revealed that a high relationship quality assessed between week 5 and week 25 of pregnancy had a positive effect on birth experience for the mother and on psychological well-being during the transition to parenthood for both mothers and fathers. Results of the retrospective quasi-experimental field study (Study 2) revealed that the continuous presence of the partner was associated with a higher probability of a low-intervention birth and a more positive birth experience. Presence of a partner for only part of the birth did not positively predict labor and birth, but did positively predict the birth experience. The effects were independent of relationship quality.

**Conclusion:**

The results of both studies highlight the importance of partners for psychological well-being during labor and birth and the transition to parenthood.

## Background

Childbirth and also the transition to parenthood are processes of change that include physical and psychological challenges, such as fatigue, physical vulnerability (e.g., loss of bodily fluid), psychological processing of labor and birth, and adjustment to the new situation and routines. If relationship quality is high, couples’ (emotional) support for each other is likely to be higher. Emotional support within the relationship could reduce potential stress, and relationship quality could function as an important buffer against stress. Literature reviews and meta-analytic studies have shown higher marital quality to be related to greater personal well-being [1], higher life satisfaction [2], and better physical health [3]. For the concept of dyadic coping (reciprocal stress reaction management) positive associations between better dyadic coping and relationship quality and stability were found [4, 5]. More specifically in relation to pregnancy and stress, research indicated that for women positive attitudes towards the partner positively affect well-being during pregnancy complications [6], thus revealing a positive effect for relationship quality on pregnancy-related stress. A recent study found correlations between antenatal mental health and relationship quality for fathers [7].

A series of studies have focused on the role of attachment for labor and birth and the transition to parenthood. Attachment theory postulates that having a secure attachment style can be a resource during stressful life events [8]. A securely attached primary attachment figure can help to reduce stress in situations perceived as threatening. Though adult attachment is not the same as the experienced attachment in childhood [9], the transition to parenthood might stimulate memories of attachment experiences, leading to an activation of attachment processes [10–12]. Accordingly, studies on attachment style and transition to parenthood have indicated associations between secure adult attachment style and less parenting stress [13], more empathy during the postpartum period [14], less depressive symptoms [10, 11], and also a positive effect on men’s trauma symptoms [11]. In the context of labor and birth, attachment style has been found to be associated with perceived birth pain [15] and with birth experience [16] in that having an anxious attachment style is associated with more pain during labor and birth and having a secure attachment style is associated with a more positive birth experience.

### Relationship quality versus presence

Integrating the available research, it becomes evident that high relationship quality operationalized as e.g., dyadic coping, attachment, or attitudes toward the partner can be a protective factor for stressful life conditions. A study unrelated to pregnancy and birth indicated a physiological stress-reducing effect (reduced cortisol and heart rate response) when there was physical contact between romantic partners prior to a stressful situation, surprisingly, the protective results were independent of relationship quality [17]. That is, the positive effect of partner support could be explained by the presence and physical contact before the stressful situation, but not by how satisfied the couples were with each other (in everyday life).

Today, in many cultures, women giving birth are routinely accompanied by their partners. Because it would be absolutely unethical to manipulate the presence of partners for experimental purposes, it is very difficult to empirically investigate the effect of the presence of the partner (or other close relationship figures) for birth. During the COVID-19 pandemic, however, many hospitals around the world excluded partners from labor and birth to reduce COVID-19-related risk. Exclusion of partners drew considerable media attention, e.g., by *The New York Times* [18] and was also criticized by the World Health Organization (WHO) [19], which advocated women should have the opportunity to choose an accompanying person for labor and birth even during the pandemic. The conceptualization of it being *nicer* to give birth with a partner present trivializes the experience. The question arises, whether the partner’s presence is merely *nice to have* – and thus dispensable during a pandemic or other crises – or whether there are substantial medical and psychological benefits for women who give birth with their partner present. Moreover, as outlined above, it is of interest whether a potential positive effect would be based on the partner’s presence or whether relationship quality would be the more important factor. Due to the special situation of the COVID-19 pandemic and our two conducted studies using a longitudinal design (covering birth and the transition to parenthood) and a quasi-experimental retrospective study, we were able to approach this question.

### The role of partners and other close relationship figures for childbirth

Frequent birth companions are the biological fathers of the babies. Research indicates that fathers do not take only a passive role during labor and birth, e.g., a recent scoping review suggested that fathers experience emotions such as anxiety and helplessness during labor and birth and are concerned about the health of the mother and child [20]. Fathers may also develop psychological symptoms after birth [11, 21]. Results of qualitative studies indicated that this is also the case for non-birthing mothers [22]. Further, it can be assumed that in a high resource country like Germany, where it is no longer culturally embedded for persons other than the partner to be present at the birth, a woman will freely choose a birth companion who is emotionally close to her and therefore emotionally involved in the birth process and concerned about the well-being of mother and child. The WHO states that women have different preferences (e.g., fathers, female relatives) regarding birth companionship [23]. This is consistent with findings that many different people such as partners, friends, and parents can act as important attachment figures for adults [8]. Thus, in the second study we chose to also include any partner and close relationship figure, regardless of if they were the biological parent. Whether different accompanying persons (biological fathers, non-biological fathers, non-birthing mothers, etc.) differ in their support is beyond the scope of the present paper and should be explored in further studies.

### The present research

The aim of this paper was to replicate previous findings indicating close relationships to be a protective factor for stressful life conditions for the two contexts (1) birth and (2) the transition to parenthood (Study 1). By doing so, we propose a model that includes different variables of the relationship quality and includes female and male data, thus dyadic data. We further (Study 2) aimed to test the specific roles of relationship quality versus the presence of the partner during stressful birth-related life events.

In Study 1 we first focused on relationship quality, which covered not only one aspect of the relationship (e.g., attachment), as has been typical in the majority of previous studies (e.g., [11, 13–16]), but instead comprised the constructs relationship satisfaction, attitude toward the partner, dyadic coping, and attachment to the partner. By including a variety of relationship qualities, we aimed to capture relationship quality more adequately than by considering just one single aspect. Criterion variables were the labor and birth process, birth experience, and the postpartum period until six weeks after the birth, which we have termed the transition to parenthood. We aimed to integrate the results into a comprehensive, fixed-reliability Single Indicator model (SI model). Due to the rather small sample size and large number of indicators per factor, we chose a fixed-reliability SI model over a conventional multiple-indicator structural equation model (SEM; see [24]. We predicted that for women higher relationship quality would positively affect both the process of labor and birth, leading to a higher probability of a low-intervention birth, and the birth experience, leading to a more positive birth experience, regardless of whether birth proceeded without intervention. Based on previous studies, women’s birth-related mindset, medical risk, and being primiparous were integrated into the model as control variables. Furthermore, we assumed that in addition to the birth experience, relationship quality would have a positive influence on the potentially stressful phase after birth, assessed with Ecological Momentary Assessment; EMA; [25], for both women and men. By using EMA it was possible to capture repeated measured data about the participant’s present emotional well-being and behavior in their natural environment and to reduce biases due to retrospective recall [25].

In Study 2, we explored whether independent from relationship quality, childbearing persons might benefit from the presence of the partner. For this purpose, we examined women who gave birth during the COVID-19 pandemic – either with or without the presence of their partners. Specifically, we tested the hypotheses that the partners’ presence at birth increases the probability of (a) a low-intervention birth and (b) a positive birth experience. We also expected a positive but smaller effect if the partner was temporarily present. These assumptions were preregistered (see OSF link below). In additional exploratory analyses we tested whether potential associations of the partner’s presence with low-intervention birth and with birth experience were moderated by relationship quality, emotional well-being experienced before birth (retrospectively assessed), and continuous midwifery support. We also aimed to conceptually replicate the SI model extracted in Study 1 (see below) and explored by whom participants, the birthing persons, felt most supported during labor and birth, and how they evaluated the partner’s support.

Data, SPSS syntaxes, the preregistration of Study 2, and additional results and materials can be found at OSF: https://osf.io/9v2y6/?view_only=23a590304c654489a543535ec021a833.

## Method

### Measurement times

Study 1 was part of a broader longitudinal study with different objectives and a variety of measurement times and variables. Data collection took place between 2016 and 2018. For reasons of readability, only the measurement points and variables relevant for this study are presented here. A complete list of all measurement points, variables, the exact order of the questionnaires, and data can be found at OSF. Measures were obtained in the first two trimesters of pregnancy (t1: e.g., relationship questionnaires), one time within the first week after birth (t2: e.g., labor and birth outcomes), and using EMA within the first six weeks after birth (postpartum adjustment). Note, most participants completed questionnaires in the first half of pregnancy. Gestational age at the first measurement time depended on how early in the pregnancy we could engage the women to participate, and varied between week five (0.7%) and week 25 (0.3%) week. The mode was week 15 (11.8%) and seven participants (2%) started between week 21 and week 25, or at the beginning of the second half of pregnancy.

Study 2 used a retrospective study design, surveying participants who gave birth during the first COVID-19 lockdown in Germany between March 15 and April 8, 2020. Data collection took place from June 29 to August 24, 2020.

For both studies, data collection took place online. For Study 1, t1 and t2 questionnaires had to be completed on the computer due to technical peculiarities. The EMA was performed via mobile phone. For Study 2, participants could choose whether they wanted to answer the questions on a computer or on a mobile phone.

### Participants

Both studies presented in this paper were conducted in Germany, using a German sample. In the first study partnered heterosexual dyads participated. In the second study, only the childbearing parent took part, and participants with and without partners were welcome to participate.

**Study 1.** For the longitudinal study, 304 cisgender, heterosexual, partnered dyads were used. Minor fluctuations in the sample size occurred depending on the time of measurement (t1: *n* = 304; t2 for females: *n* = 293, t2 for males: *n* = 279; EMA for females: *n* = 293, average response rate: 74%, EMA for males: *n* = 292, average response rate: 68%). At t1 the mean age for the 304 female participants was 30.30 years (*SD* = 3.99) and for male participants was 32.58 years (*SD* = 4.51). The majority of couples were married (62.2%) at the beginning of data collection (t1). Only 3.6% indicated a length of relationship of less than a year, and the length of relationship for the remaining sample varied between one year (3.9%) and 20 (1.0%) years with a mode of six years (9.2%; the length of relationship was also assessed at t1). The vast majority of fathers were present at the birth (97.2%). Prior to data collection women completed a screening questionnaire to assess exclusion criteria. Participants being pregnant with more than one child, artificial insemination, and more than one abortion and/or more than one stillbirth in the past could not take part in the study to avoid unnecessary burden for them. Participants also had to have mobile internet access and be older than 18 years and younger than 38 years. The use of psychotropic drugs was also an exclusion criterion. Women were recruited either by the help of midwives and gynecologists or via Facebook groups and Facebook advertisement. Participating women received 100 euros and participating men 80 euros (as they had to complete fewer questionnaires) as monetary compensation. Incentives were paid after the last measurement time point eight weeks after the birth. Participants were also paid if a measurement point was missed. For the additional assessment point six months after birth no incentive was paid.

**Study 2.** The second study was completed online by 1,160 participants. Participants were recruited mainly through social networks such as Facebook and Instagram. The survey was accessible from June 29 to August 24, 2020. For 180 of the 1,160 participants, preregistered exclusion criteria were met: they did not give birth between March 15 and April 8, 2020 (the period of the official lockdown due to the COVID-19 pandemic in Germany), gave birth in out-of-hospital settings, were men, and/or indicated their data should not be used. Thus, 980 participants (979 female, 1 third gender; *M*age = 31.90 years, *Sd*age = 4.15 years) remained in the sample. Our target sample size of 250 was clearly exceeded but in favor of power maximization we retained the sample that remained after exclusion based on the defined criteria above.

### Measures used in study 1

**Measurement time 1 (t1).** The measures described immediately below were collected at t1, i.e., between week 5 and 25 of pregnancy.

*Relationship attachment.* We used the partner-specific [26] German version of the Relationship Questionnaire (RQ; [27]) to assess relationship attachment. The scale contains one prototypical description for each of the four attachment styles (secure, anxious, preoccupied, dismissive), and participants responded to each description by rating to what degree it describes themselves on a six-point Likert scale ranging from 1 = *strongly disagree* to 6 = *strongly agree* (the original answer format was changed for the present study). The responses to the insecure attachment descriptions were recoded and aggregated with secure attachment such that a high score of relationship attachment indicates secure attachment. Cronbach’s α was 0.60 for female participants and 0.59 for male participants.

*Attitudes toward romantic partner.* For measuring (explicit) attitude toward the romantic partner, we used the scale developed by Banse and Kowalick [6]. Participants were asked to answer 15 items about their partner (e.g., *I feel good when I am close to my partner*) on a six-point Likert scale ranging from 1 = *strongly disagree* to 6 = *strongly agree* (the original answer format was changed for the present study). Cronbach’s α was 0.83 for both female and male participants.

*Relationship satisfaction*. The German version [28] of the Relationship Assessment Scale (RAS; [29]) was used to assess relationship satisfaction. The scale consists of seven items (e.g., *In general, how satisfied are you with your relationship?*) that participants answered on a six-point Likert scale. The scale’s endpoint labels depended on the particular question. Cronbach’s α was 0.87 for female and 0.82 for male participants.

*Dyadic coping.* Dyadic coping was measured with the first two subscales of the Dyadic Coping Inventory (DCI; [30]). The first subscale comprises four items about the desired involvement of the partner when feeling stressed or burdened (e.g., *I ask my partner to take over tasks and activities if I am overloaded*). The second subscale contains 11 items assessing the partner’s reaction to the expressed stress (e.g., *She/he gives me the feeling that she/he understands me and that she/he is interested in my stress*). The subscales were combined to a single score of dyadic coping, as is also suggested in the test manual [30] and our study did not aim to obtain precise information about certain coping difficulties. All items were answered on a six-point Likert scale (1 = *strongly disagree* and 6 = *strongly agree*). Cronbach’s α was 0.87 for female and 0.88 for male participants.

*Birth-related mindset.* Birth-related mindset was assessed using the Mindset and Birth Questionnaire (MBQ; [31]), which consists of 18 items and four subscales (for the present study an overall score was used). The scale measures trust in midwives versus doctors, birth-related shame and disgust sensitivity, the participant’s view of drug support and vaginal birth. The answer format is a six-point Likert scale ranging from 1 = *strongly disagree* to 6 = *strongly agree*. Cronbach’s α for this questionnaire in Study 1 was 0.89.

**Measurement time 2 (t2).** Low-intervention birth and birth experience were assessed shortly (within the first week) after birth at t2.

*Low-intervention birth.* To summarize the complex process of labor and birth we combined (effect-coded) different birth variables into one variable indicating whether participants had a low-intervention birth (= 1) or not (= -1). The variable low-intervention birth [32] is adapted from the normal birth index [33]. If labor and birth was induced (19.9%), or augmented during the process (33.3%), an epidural (24.7%), or episiotomy (13.9%) was performed, and/or the birth was ended by vacuum or forceps (9.4%) or C-section (17.0%), the birth was counted as a high-intervention birth. The numbers in parentheses refer to the frequencies of the specific interventions in the present sample. If none of the mentioned interventions were performed the birth counted as a low-intervention birth, which was the case for 39.7% of the participating women. The C-section rate was lower than would be expected for the years 2016–2018, at 17.0% compared to 29.1–30.5% in the German population, but the number of vaginal assisted births was minimally higher (about 6% on population level compared to 9.4% in the present study; [34–36]; and no population-level prevalence is available for the other interventions. Note, potential problems with using this binary index of low intervention are addressed in the discussion.

*Birth experience.* To assess the participants’ general satisfaction with the birth experience (e.g., *I would wish for another birth like this.*), we used the Birth experience scale [31]. The scale consists of 10 items answered on a six-point Likert scale ranging from 1 = *strongly disagree* to 6 = *strongly agree*. Cronbach’s α in the present study was 0.90 for women and 0.85 for men.

**Postpartum adjustment.** For assessing postpartum adjustment within the first six weeks after birth we used EMA [25], which yielded repeated-measures data about current emotional states or behavior in participant’s natural environments [37]. In the EMA process, participants received a link to an online questionnaire on their mobile phones at a random time of a day (time-based sampling; [37]). Links were sent daily in weeks one and two, and weekly in weeks three through six, all between 9am and 8pm. The questionnaire contained questions about participant’s emotional and general well-being, and the perceived infant’s well-being (see below). For determining Cronbach’s α, all measurement times were divided into split halves (odd-even).

For measuring emotional well-being, we used 12 items from the Quality of Life Profile for Chronically Ill Patients [38]; women: α = 0.93, men: α = 0.96), and for general well-being two (men) and three (women) items, respectively, measuring how pain-free (answered only by women), healthy/fit and resilient participants felt (women: α = 0.95, men: α = 0.95). Both scales were answered on a six-point Likert scale ranging from 1 = *strongly disagree* to 6 = *strongly agree*. The baby’s perceived well-being was assessed with six items using a semantic differential (six points). Items measured the crying and sleeping behavior of the baby, how satisfied, exhausted, and quiet the baby appeared, and how easy the baby could be comforted (women: α = 0.88, men: α = 0.86).

**Being primiparous and medical risk.** Analogous to previous studies (e.g., [31, 32]), giving birth for the first time (primiparous: 54.9% in the present study) and having an identified medical risk were treated as control variables. The questions concerning participants’ prenatal risks were based on the German maternity guidelines [39]. They assessed e.g., previous C-sections, fetal malposition, and health status of the mother. The questions were answered with yes or no according to the presence or absence of the risk factor. If one or more risks were present (true for 45.3% of participants in Study 1), the variable risk was coded.

### Measures and procedure used in study 2

As in the first study, the variables relationship attachment (α = 0.62), attitudes toward the romantic partner (α = 0.88), relationship satisfaction (α = 0.88), birth-related mindset (α = 0.83), being primiparous (51.1%), medical risk (55.6%), low-intervention birth (30.9%; induction: 26.3%; augmentation during labor and birth: 25.6%; epidural: 34.2%; episiotomy: 14.5%; assisted vaginal delivery: 8.3%; C-section: 25.7%), and birth experience (α = 0.93) were assessed. For medical risk, in this study we also asked for a SARS-CoV-2 diagnosis at the time of delivery, and four participants had tested positive. Note again the C-section rate in our study was lower than for the German population, which was 29.5% in 2020, and the rate of vaginal deliveries in this study (8.3%) was slightly higher than typical for the German population, which was 6.4%% in 2020 [40].

**Partner’s presence.** Participants were asked to indicate who was planned to accompany them during labor and birth (options: male romantic partner, female romantic partner, other), whether the partner was able to be present, and if so, to what extent (the entire time or temporarily, i.e., during active delivery stage). The majority of the sample (95.1%) had planned for their male partner to accompany them, 1.0% for their female partner, and 3.9% for someone else.^1^ In 500 cases (51.0%), the companion was continuously present, in 407 (41.5%) temporarily, and in 73 cases (7.4%) not at all. Based on the participants’ answers, we generated the two dummy coded variables, *presence* (1 = partner continuously present at birth or partner temporarily present at birth and 0 = partner not present) and *continuous presence* (1 = partner continuously present at birth and 0 = partner temporarily present at birth or partner not present) to conduct further analyses. This procedure makes it possible to test the effect of all possibilities (continuously present, temporarily present, not present at all) on low-intervention birth and on birth experience.

**Emotional well-being before birth.** To retrospectively assess the participants’ emotional well-being in the days before birth, we again used 10 items of the Quality of Life Profile for Chronically Ill Patients [38], using the following instruction: *We would like you to recollect the last few days before giving birth. How were you feeling when thinking of the imminent birth?* The answer format for the items was again a six-point Likert scale ranging from 1 = *strongly disagree* to 6 = *strongly agree*. Cronbach’s α in the present study was 0.91.

**Perceived support.** In Germany it is possible to hire a freelancing midwife for labor and birth. The advantage over the standard care by a hospital midwife is continuous one-to-one care. For the accompaniment, however, extra costs must be paid and the number of freelancing midwives providing this type of care is limited. To assess one-on-one support, we asked what kind of midwifery support participants were given during labor and birth with the following options: freelancing midwife, hospital midwife, and no midwife. For the analyses the variable was dummy coded. In the present study 12.7% of the participants had access to one-on-one support from a midwife.

We also assessed the participants’ perception of different support forms originating from different support sources. The different support forms were based on Hodnett [41] and included emotional support, comfort measures, information, and advocacy. Participants were asked to rate the perceived support on the four items for freelancing midwives (if present), hospital midwives, other hospital staff, and the partner/other accompanying person (if at least temporarily present) on a six-point Likert scale ranging from 1 = *not at all* to 6 = *very much*. This allowed us to compare the participants’ perception of their partners’ support on different levels to their perception of the medical staff’s support.

**Procedure.** The second study was conducted online. Participants were first informed that their participation was voluntary, anonymous, and could be ended at any time. Then they were presented with questions concerning their age and gender. To minimize a potential influence of the birth experience on the birth-related mindset, participants first completed the MBQ before continuing with the birth-related questions, including those concerning the different prenatal risks, the planned companion, and their companion’s presence. They then completed the Quality of Life Profile and the Birth experience scale, followed by the questions concerning participants’ perception of the different supporters and the different support forms. The relationship questionnaires were answered last, and only by those who had at least planned for their partner to accompany them. In the end, all participants could indicate whether their data should be used or not, leave a personal remark, and share their e-mail address if they wanted us to send them a summary of the results. In this case, the address was registered separately.

### Areas under the curve, and ***p***-value

As areas under the curve (AUC) provide better evidence of the strength of associations with a dichotomous outcome than correlation coefficients do (especially if low base rates are expected) [42], we use AUCs instead of correlation coefficients in our analyses. AUCs indicate with what probability the value of a continuous or dichotomous variable can be assigned to the value of a second dichotomous variable. Value of AUCs vary between 0 and 1. An AUC of 0.50 indicates a random effect, and AUCs close to 1 (similar to a positive correlation) or 0 (similar to a negative correlation) indicate a perfect prediction. An AUC of ≥ 0.64 (or ≤ 0.39) is considered a moderate effect and an AUC of ≥ 0.71 (or ≤ 0.29) a large effect [42]. To facilitate readability, we set the *p*-value to 0.01 for all reported results if not stated otherwise.

## Results

We will first briefly discuss descriptive statistics and intercorrelations as well as the relationship variables’ associations with birth, birth experience, and postpartum well-being for Study 1. The results are also presented in Tables 1, 2 and 3. Readers in a hurry may want to proceed directly to the comprehensive SI model, presented in paragraph *Single Indicator model.* We then proceed with the results of Study 2 to test the potential positive effect of partner’s presence at birth. For Study 2, descriptive statistics, AUCs, and zero-order correlations of the key variables are displayed in Table 4. Associations of low-intervention birth with birth experience and with the partner’s presence were revealed and further tested in the pre-registered *main analyses*.

**Table 1:** Descriptive statistics and zero-order correlations of the relationship measures (Study 1)

	*M*	*SD*	1	2	3	4	5	6	7	8	
Mothers											
1. Attachment	5.20_ A_	0.77	(0.60)	0.52**	0.57**	0.57**	0.21**	0.19**	0.22**	0.19**	
2. Explicit attitudes	5.48	0.45		(0.83)	0.72**	0.65**	0.33**	0.28**	0.36**	0.33**	
3. Relationship satisfaction	5.33_ A_	0.62			(0.87)	0.64**	0.34**	0.32**	0.39**	0.37**	
4. Dyadic coping	4.86	0.68				(0.87)	0.29**	0.23**	0.25**	0.33**	
Fathers											
5. Attachment	5.12_ A_	0.79					(0.59)	0.58**	0.59**	0.43**	
6. Explicit attitudes	5.39	0.50						(0.83)	0.78**	0.49**	
7. Relationship satisfaction	5.30_ A_	0.58							(0.82)	0.57**	
8. Dyadic coping	4.53	0.74								(0.88)	

*Note. N* = 304, **p < .01, *p < .05. If indexed with _A_ mothers and fathers were not statistically different from each other (*p* < .01). Reliability (Cronbach’s α) in parentheses.

### Descriptive statistics and intercorrelations in study 1

Means and standard deviations for each of the utilized measures are displayed in Tables 1 and 2. Additionally, we performed paired *t*-tests to test for sex differences. Results indicated female and male participants did not differ in attachment, implicit attitudes, relationship satisfaction, or in their evaluation of the infant’s well-being and behavior. These results are also presented in the tables and non-significant mean differences are marked with an index. Zero-order correlations between the relationship measures can be found in Table 1. As expected, strong correlations occurred within the sexes, indicating an overlap in the measured constructs and thus a latent attribute. Correlations between the sexes were small to medium. Table 2 shows the intercorrelations of birth experience and the variables assessed during the first six weeks after birth. Within the sexes correlations were medium to high, but between the sexes correlations were rather small except for strong correlations for birth experience and perceived infant’s well-being.

**Table 2:** Descriptive statistics and zero-order correlations of birth experience and postpartum adjustment variables (Study 1)

	*M*	*SD*	1	2	3	4	5	6	7	8
Mothers										
1. Birth experience	4.79	1.02	(0.90)	0.38**	0.32**	0.24**	0.60**	0.18**	0.24**	0.19**
2. General well-being	4.09	0.83		(0.95)	0.68**	0.43**	0.19**	0.12*	0.20**	0.22**
3. Emotional well-being	5.17	0.52			(0.93)	0.53**	0.13*	0.22**	0.31**	0.28**
4. Infant’s well-being	4.77_ A_	0.61				(0.88)	0.18**	0.14*	0.17**	0.54**
Fathers										
5. Birth experience	5.20	0.76					(0.85)	0.25**	0.32**	0.30**
6. General well-being	4.73	0.82						(0.95)	0.75**	0.37**
7. Emotional well-being	5.30	0.50							(0.96)	0.36**
8. Infant’s well-being	4.74_ A_	0.58								(0.86)

*Note. N* varied between 274 and 290. **p < .01, *p < .05. If indexed with _A_ mothers and fathers were not statistically different from each other (*p* < .01). Reliability (Cronbach’s α) in parentheses.

### Associations of relationship variables with birth, birth experience, and postpartum well-being in study 1

Table 3 displays the associations between the relationship variables and low-intervention birth, birth experience, and postpartum well-being. Results did not indicate significant associations between relationship variables and low-intervention birth or the birth experience for women or for men. The only exception was a significant correlation between female dyadic coping and birth experience, indicating dyadic coping increased the probability of a positive birth experience. However, the effect was small. Small to medium correlations emerged between the relationship variables and the variables general and emotional well-being such that better relationship quality was associated with better well-being after birth. Aside from a small positive correlation between female attachment and more positively perceived well-being and behavior of the infant, relationship variables were not associated with the infant’s well-being and behavior.

**Table 3:** AUCs and partial correlations for/between the relationship variables and low-intervention birth, birth experience, and the variables assessed postpartum (EMA; Study 1)

	α	Low-i. birth^A^	Birth exp.	General well-being	Emotional well-being	Infant’s well-being
Mothers						
Attachment	0.60	0.52	0.09	0.27**	0.36**	0.13*
Explicit attitudes	0.83	0.50	0.11	0.11	0.23**	0.02
Relationship satisfaction	0.87	0.49	0.08	0.16**	0.29**	−0.04
Dyadic coping	0.87	0.51	0.14*	0.18**	0.29**	0.08
Fathers						
Attachment	0.59	0.48	0.02	0.20**	0.31**	0.03
Explicit attitudes	0.83	0.49	0.01	0.32**	0.33**	0.07
Relationship satisfaction	0.82	0.45	0.02	0.36**	0.38**	0.10
Dyadic coping	0.88	0.46	−0.05	0.20**	0.28**	0.08

*Note.* T1: *n* = 304, t2 for mothers: *n* = 293, t2 for fathers: *n* = 279. If indexed with ^A^ the variables are assessed in AUC; all other relationships are correlation coefficients. **p < .01, *p < .05.

In sum, the results of the AUCs and correlation analyses did not suggest that the couple’s relationship quality impacts labor and birth or the birth experience as we had expected. However, we nevertheless included the variables in the a priori hypothesized SI model (see paragraph *Model assumptions*).

### Single indicator model for study 1

As outlined above we estimated a fixed-reliability SI model to test our assumptions in one comprehensive model. The mean of the scales relationship attachment, attitudes toward romantic partner, relationship satisfaction, and dyadic coping was used as an indicator of relationship quality (high scores indicate a higher quality). The reliabilities of the scores used as indicators were fixed to 0.90 for all variables. Due to the dichotomous coding of the dependent variable low-intervention birth, WLSMV was chosen as the estimator. Analyses were performed in Mplus 7.4 [43] using the default convergence criteria and the default processing of missing values. Co-variances of all exogenous variables as well as covariances between the residuals of the endogenous variables were freely estimated. We assessed the model fit using the χ²-test (α = 0.05) and the fit indices RMSEA (≤ 0.05), CFI (≥ 0.96), and WRMR (≤ 1.00), with the recommended cutoff values in parentheses [44].

**Fig. 1:**
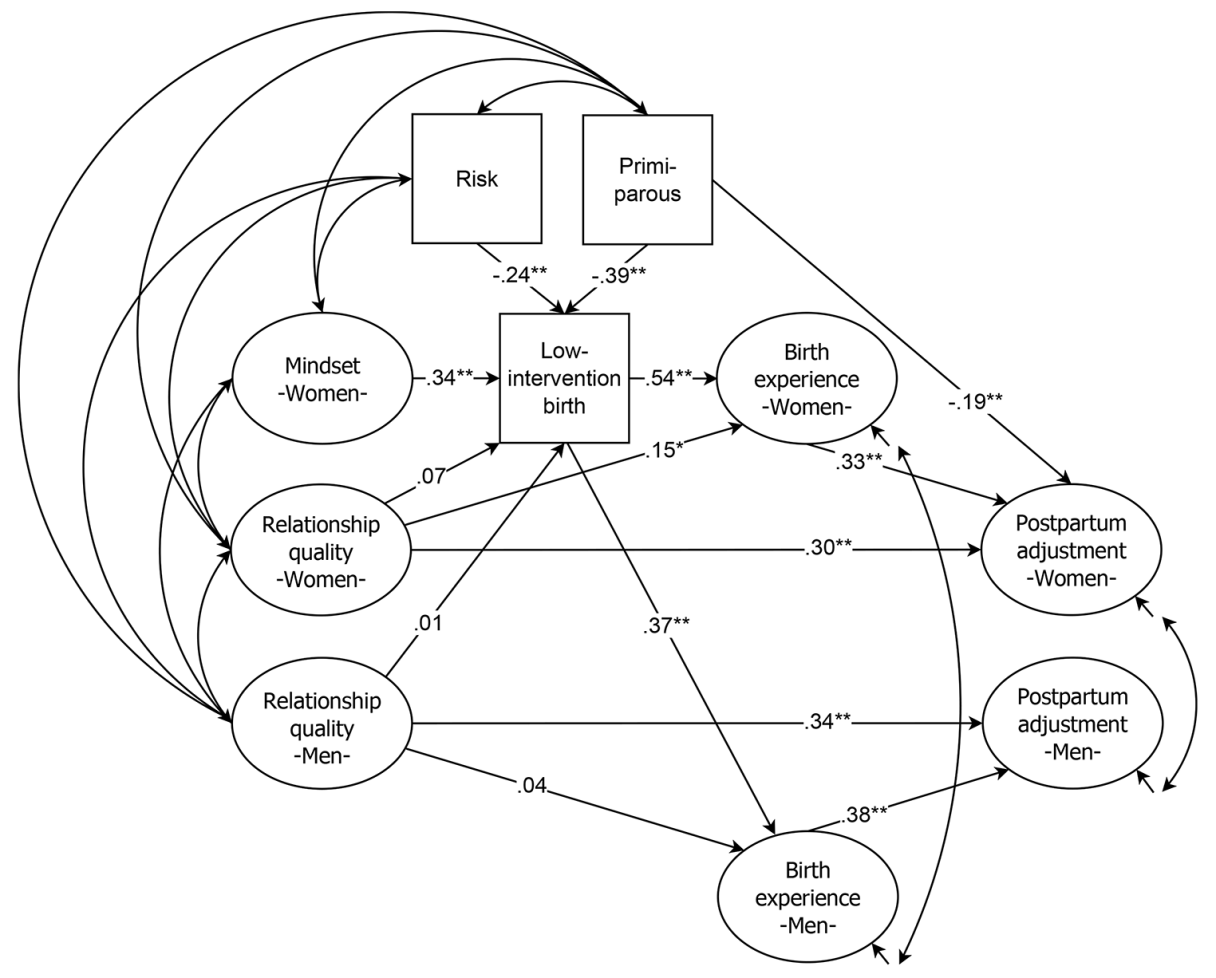
Linear structure of the latent (displayed as ovals) and manifest (displayed as squares) variables with the standardized weights of the SI model (Study 1). Note: **p < .01, *p < .05

In Fig. 1 the linear structure of the latent (displayed as ovals) and manifest (displayed as squares) variables of the SI model with the standardized weights is presented. The χ²-test of model fit was not significant (χ² = 16.795, *df* = 19, *p* = .604) and the approximate fit indices (RMSEA = 0.000, CFI = 1.000, WRMR = 0.366) also supported the good fit of the model [44]. The model displays three significant predictors of low-intervention birth: prenatal risk (-0.24) and being primiparous (-0.39) decreased the probability of a low-intervention birth, having a more natural birth-related mindset during pregnancy (0.34) increased the probability of a low-intervention birth. Contrary to our initial assumption, but as already indicated by the AUC analyses, low-intervention birth was not predicted by the women’s nor by the men’s ratings of relationship quality. Results revealed that low-intervention birth positively predicted both women’s (0.54) and men’s (0.37) birth experiences (see also [32]). Women’s better relationship quality was associated with a more positive birth experience. However, the effect was relatively small (0.15, *p* < .05). Female postpartum adjustment was negatively related to being primiparous (-0.19), positively related to a positive birth experience (0.33), and also positively predicted by female relationship quality (0.30). Accordingly, male postpartum adjustment was also predicted by male relationship quality (0.34).

Thus, results of the SI model indicated that relationship quality was not associated with an increased prevalence of low-intervention birth but did positively predict birth experience for female participants, and postpartum experience for both genders.

### Main analyses of study 2^2^

When testing our main hypothesis in our second study, we conducted – as preregistered – logistic regression analyses (not alpha-adjusted) for the dependent variable low-intervention birth using the control variables medical risk, being primiparous, birth-related mindset, and the two variables presence and continuous presence as predictors. Results indicated all three control variables significantly (all *p*s < .001) predicted low-intervention birth, risk: *B* = -1.396, *SE* = 0.173; being primiparous: *B* = -1.752, *SE* = 0.177; mindset: *B* = 1.030, *SE* = 0.134. Contrary to our predictions only continuous presence, *B* = 0.618, *SE* = 0.172, *p* < .001, and not temporary presence, *B* = 0.423, *SE* = 0.365, *p* = .246, predicted the outcome low-intervention birth. That is, only if the attachment figure was continuously present during labor and birth was their presence associated with low-intervention birth. Odds ratios for the above regression coefficients were 1.86 for the contrast between continuous and partial presence, and 1.53 for the comparison of partial presence with absence.

For the dependent variable birth experience, we conducted linear regression (again not alpha-adjusted) using low-intervention birth and the variables presence and continuous presence as predictors. In line with our hypothesis, low-intervention birth, *B* = 0.825, *SE* = 0.084, *p* < .001, and both presence, *B* = 0.438, *SE* = 0.151, *p* = .004, and continuous presence, *B* = 0.533, *SE* = 0.081, *p* < .001, positively predicted birth experience. The effect size of continuous versus partial presence was *d* = 0.41, and the effect size of partial presence versus absence was *d* = 0.34 (*d* adjusted according to Borenstein, [45].^3^

### Exploratory data analyses for study 2

**Fig. 2:**
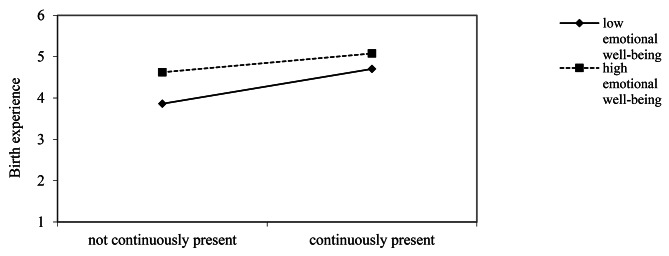
Moderation analysis: Birth experience as a function of emotional well-being before birth and the partner’s presence during labor and birth (Study 2)

**Moderation analyses.** We conducted hierarchical multiple regression analyses [46] to test whether emotional well-being experienced before birth (retrospectively assessed) moderated the associated between the partner’s continuous presence during labor and birth and birth experience.^4^ The regression of the birth experience on continuous presence and emotional well-being (*z*-standardized) resulted in a significant outcome, *R*^2^ = 0.13, *F*(2, 977) = 73.27, *p* < .001. Adding the interaction term of the two predictors, the amount of variance explained was significantly increased, *ΔR*^2^ = 0.01, *p* = .014. In the final model, the significant interaction effect, *β* = − 0.10, *p* = .014, was accompanied by two main effects, continuous presence: *β* = 0.25, *p* < .001, and emotional well-being: *β* = 0.29, *p* < .001. Thus, new mother’s the evaluation of the birth experience also depended on their emotional well-being before birth. The effect was weaker for non-continuous presence than for continuous presence, but was strengthened by the retrospectively assessed well-being before birth (Fig. 2).

**Fig. 3:**
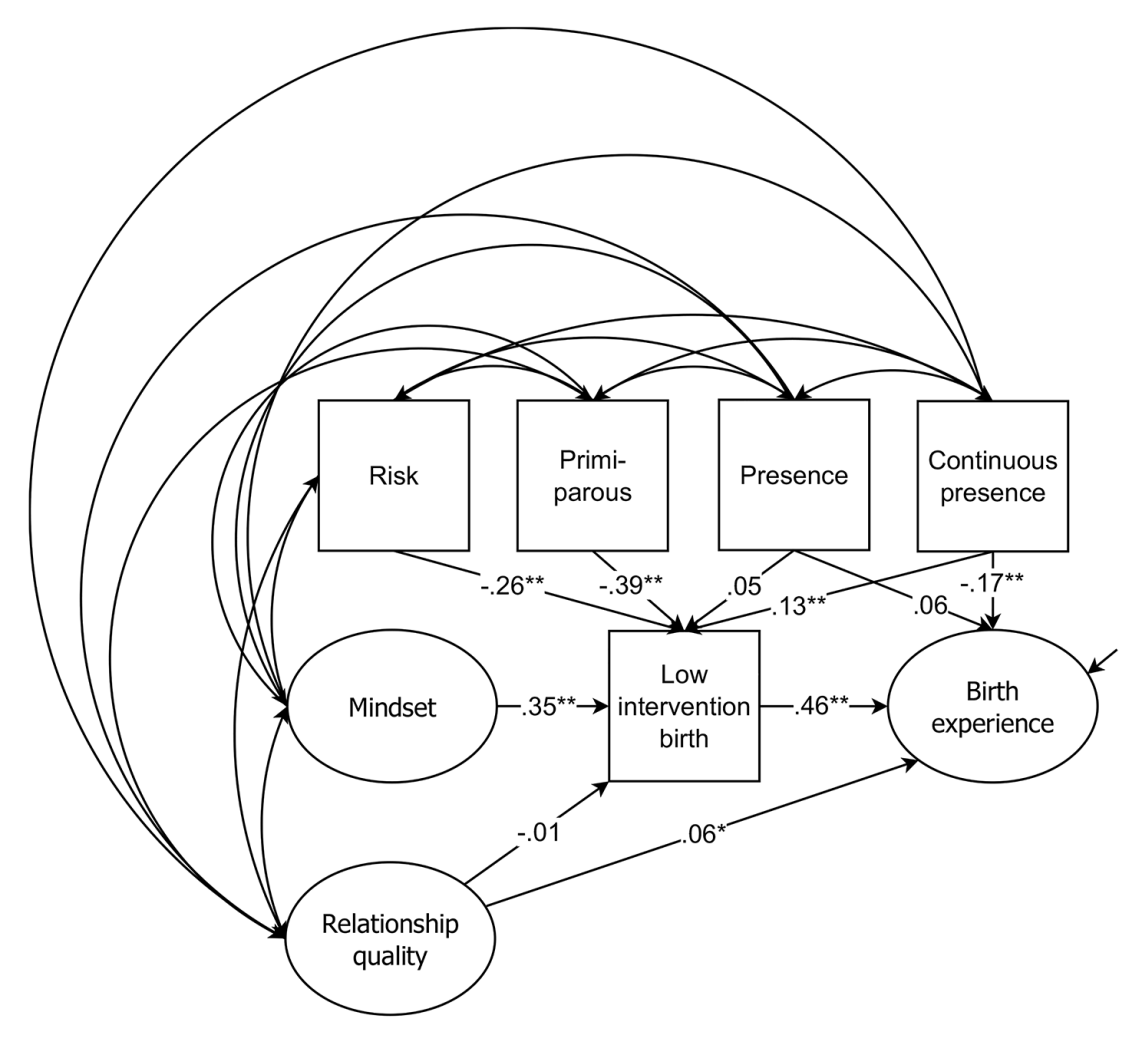
Linear structure of the latent (displayed as ovals) and manifest (displayed as squares) variables with the standardized weights of the SI model (Study 2). Note: *N* = 980. **p < .01. *p < .05

**Single indicator model.** We also explored whether the results could be integrated into an SI model again. As in Study 1 the relationship scales relationship attachment, attitudes toward the romantic partner, and relationship satisfaction comprised relationship quality. Again, for all variables reliabilities were fixed to 0.90, WLSMV was chosen as the estimator, default convergence criteria and processing of missing value of Mplus 7.4 was used, and co-variances of all exogenous variables and covariances between the residuals of the endogenous variables were freely estimated. In Fig. 3 the linear structure of the latent (displayed as ovals) and manifest (displayed as squares) variables of the SI model with the standardized weights is revealed. The new model both replicates the model from Study 1 (note though the effect of relationship quality on birth experience was weaker than in Study 1) and the results of the previously conducted regression analyses of this study, with the difference that partner’s presence had no significant effect on birth experience. The χ²-test of model fit was significant (χ² = 8.552, *df* = 19, *p* = .036), however, the approximate fit indices (RMSEA = 0.043, CFI = 0.992, WRMR = 0.323) indicated a good fit of the model [44].

**Perceived support.** Table 5 shows the means and standard deviations as well as the results of paired *t*-tests for the participant’s perception of different support forms (emotional support, comfort, information, and advocacy) originating from the different support sources (close attachment figure and maternity caregivers). Results were calculated separately for the two groups: participants with one-on-one support by a freelancing midwife and participants without such support. Results indicated that except for the variable information, the partner provided the strongest support in both groups. In the group with one-on-one support from a freelancing midwife, results revealed that the support provided by the freelancing midwife was rated significantly higher than that provided by the clinic midwife. However, the results do not provide any information on whether this was due to a greater relationship of trust with the freelancing midwife, or simply because in these cases the care was provided mainly by the freelancing midwife.

**Table 4:** Descriptive statistics, AUCs, and zero-order correlations of the key variables (Study 2)

	freq.	*M*	*SD*	1	2	3	4	5	6	7	
1. Low-intervention birth	30.9%	-	-	-	0.74**	0.57*	0.59**	0.39**	0.35**	0.70**	
2. Birth experience	-	4.58	1.30		(0.93)	0.66**	0.67**	0.45*	0.37**	0.23**r	
3. Presence	92.6%	-	-				-	0.48	0.52	0.60**	
4. Continuous presence	51.0%	-	-				-	0.46*	0.47	0.60**	
5. Risk	55.6%	-	-						0.44**	0.45*	
6. Being primiparous	51.1%									0.42**	
7. Mindset	-	4.50	0.72							(0.83)	

*Note. N* = 980. **p < .01, *p < .05. ^r^Correlation coefficient, all other values are AUCs. Reliabilities (Cronbach’s α) in brackets.

**Table 5:** Descriptive statistics of the variables measuring the perceived support of the partner and different maternity caregivers (Study 2)

	Emotional support		Comfort		Information		Advocacy		
	*M*	*SD*	*M*	*SD*	*M*	*SD*	*M*	*SD*	
**Participants with 1:1 support of a freelancing midwife** (*n* = 109)									
Partner	5.58_0_	0.874	5.29	1.157	3.37_AB_	1.859	5.18_0_	1.334	
Freelancing midwife	4.81_0_	1.630	3.78	1.939	4.59_000_	1.744	4.58_0_	1.857	
Hospital midwife	3.73_ A_	1.989	2.95	1.912	3.56_A0_	2.025	3.40_ A_	2.104	
Other clinic staff	3.50_ A_	1.889	2.44	1.756	3.14_B0_	2.057	3.20_ A_	2.013	
**Participants without 1:1 support of a freelancing midwife** (*n* = 770)									
Partner	5.57_0_	0.837	5.21	1.275	3.35_C0_	1.847	5.07_0_	1.407	
Hospital midwife	4.84_0_	1.434	3.64	1.828	4.42_00_	1.577	4.29_0_	1.695	
Other clinic staff	3.88_0_	1.737	2.58	1.782	3.37_C0_	1.862	3.34_0_	1.876	

*Note.* If in a row means are indexed with a letter, they are not statistically different from each other (*p* < .05). Results were calculated separately for the two groups participants with and without 1:1 support by a freelancing midwife.

## Discussion

In the present paper we introduced data on the importance of partners and relationship quality for birth, birth experience, and the transition to parenthood using evidence from a longitudinal (Study 1) and a quasi-experimental study (Study 2). Results from Study 1 demonstrated the positive effect of relationship quality on stressful life events for both women and men. However, this effect became especially evident for the phase of transition to parenthood. For this transition, both women and men benefited from greater relationship quality. Men and women who reported greater relationship quality also reported better general and emotional well-being compared to men and women who reported lower relationship quality. The results are consistent with previous research that has investigated mostly single aspects of relationship quality and their associations with the transition to parenthood (e.g., [10, 11, 13]). Relationship quality was only weakly positively related to birth experience, replicating previous research that found a positive association between having a secure attachment style and a positive birth experience [16]. However, in Study 1 the effect was small and relationship quality was not found to be related to the process of labor and birth at all. Previous research has suggested that for women physical contact with their partners right before being exposed to a stressor reduced cortisol and heart rate response, independent of relationship quality [17]. This could imply that the presence of the partner could have a positive effect on birth and birth experience and that in this case relationship quality only plays a subordinate role. Since in the first study only eight fathers were not present at birth, we refrained from testing this hypothesis with the available data set of Study 1 but conducted Study 2 to explore the question.

Results of Study 2 indeed demonstrated that regardless of relationship quality the presence of the partner was positively related to low-intervention birth and to a more positive birth experience. The effect of the partner’s presence was strongest when the partner was continuously present. This effect may be because at the beginning of labor women are often alone in the delivery room. A close person can thus provide useful support and thereby reduce the likelihood of interventions such as epidurals and, most importantly, their presence increases the likelihood of a positive birth experience. Having a positive birth experience can, in turn, have a positive effect on subsequent psychological well-being [32]. Future studies should explore potential modes of action for the effect found here, including the role of physical contact (see [17]). Due to the retrospective design of Study 2, the results are mute to causality. To approach the question of causality, a longitudinal design would be necessary, but difficult to realize. During non-pandemic situations, selection effects are unavoidable because families decide for themselves whether the birth should be accompanied by the partner or not.

The exploratory moderation analysis in Study 2 suggested that for participants who reported a lower well-being prior to birth, not having the continuous presence of their partners during birth was related to a more negative birth experience than for participants who reported higher before-birth well-being and also lacked continuous presence. It is possible that women felt unwell prior to birth because they were aware they might need to give birth without their partners. For individuals for whom the prospect of birthing without the partner present was particularly distressing, this led to a lower well-being prior to birth and the absence of the partner was perceived as very negative during labor and birth, resulting in a more negative rating of the birth experience. However, since the quality of life was retrospectively assessed it cannot be ruled out that current well-being biased the perception of well-being prior to birth. Indeed, psychological research indicates memory to be subject to biases [47].

All in all, the findings of both Study 1 and Study 2 demonstrate that partners hold more than a purely passive role during labor and birth and therefore underscore the WHO’s [19] demands that women have the opportunity to be supported by a primary attachment figure (e.g., the partner) of their choice during labor and birth.

### Theoretical implications

As outlined, previous research has indicated that characteristics of high-quality relationships are positively associated with better psychological well-being (e.g., [1]). The assumption that a positive effect of relationships for coping with labor and birth only becomes evident if relationship quality is high seems rather plausible. However, the results of our studies suggest relationship quality may hold no or only a subordinate role for labor and birth, given that in both studies relationship quality did not predict labor and birth and the effects of relationship quality on birth experience were also only small. However, the effect of relationship quality became evident on the transition to parenthood (Study 1). The question arises, how do birth and the transition to parenthood differ? We argue that while birth refers to a rather short event with a clear objective (similar to the study by Ditzen et al., [17]), the transition to parenthood is a less defined event. Over the course of labor and birth it is also clear which needs are most important: those of the mother and the child. During the longer period of the transition to parenthood, the needs of the non-birthing parent become increasingly relevant again, which may create more potential for conflict. Consequently, during the transition to parenthood it might be more important that couple conflicts are resolved, both partners’ needs are addressed, and adequate dyadic coping mechanisms are applied – and these are all characteristics of high-quality relationships. Further studies should systematically test these hypotheses to gain a more comprehensive knowledge of the potential differential effects of a partner’s presence and relationship quality on stressful life events.

### Practical implications

As outlined above, the WHO [19] advocated women should have the opportunity to choose an accompanying person for labor and birth even during a pandemic (and even with a COVID-19 diagnosis). Though we cannot draw strong causal conclusions from our study, we would argue that the results nevertheless provide support for the statement of the WHO. Our results indicate a positive relationship between the partner’s continuous presence and both the birth process and birth experience. Thus, one might conclude that the partner’s presence is not *a nice to have* but rather *a must-have* and the enforced absence of partners represents a disadvantage for childbearing persons. Even without this strong rhetoric, with the current state of knowledge the exclusion of partners from labor and birth does not seem advisable, because it cannot be ruled out that the exclusion might have negative long-term consequences on health and well-being. Previous studies have revealed that labor and birth and the birth experience can have an impact on the development of psychopathological symptoms of parents [48], ([32], as well as on parent-child attachment [49, 50], [32]. Combining these findings with those of the present studies, we would argue the partner’s presence at birth should be valuable and considered a positive factor for the birth process. Further, the results of our first study, demonstrating the importance of relationship quality for transition to parenthood, could be used in practice to provide couples with approaches for strengthening the quality of their relationship already during pregnancy to ease the transition to parenthood.

### Strengths and limitations

Both Study 1 and Study 2 provide new data and insights into the role of close relationship figures, usually partners, in childbirth and the transition to parenthood using large samples (*N* = 304 dyads in Study 1 and *N* = 980 in Study 2). A recent editorial called for more studies using dyadic approaches for studying birth and the transition to parenthood [51]. Study 1 has the strength of being based on dyadic data. Since it was a longitudinal study, initial conclusions about the direction of the effect can also be made: relationship quality affects the transition to parenthood. The extent to which the effect also holds in reverse and/or whether there are third variables that better explain the found correlation needs to be explored in additional studies. The research question addressed in Study 2 is, to our knowledge, the first to compare birth outcomes and experiences while considering the partner’s presence at labor and birth in a quasi-experimental setting. Due to the correlative design results are mute to causality and, of course, a randomized controlled design would be methodologically superior and allow understanding of causality. However, as stated in the introduction, in settings in which women are free to choose attending persons for social support during labor and birth, it would be morally unacceptable to conduct a randomized controlled design. Thus, the design chosen in the present study is the most controlled approach plausible.

For both studies, the occurrence of possible selection biases and the use of the low-intervention birth index should be critically noted. As described in the method section, both studies were posted on social media channels and were thus not accessible to all pregnant and birthing women. For Study 2, which was retrospective, it is likely that women with negative birth experiences and who were particularly upset about the partner’s absence participated in the study. However, when considering the variation in the birth experience distribution, this possible effect is to be regarded as rather small. It must also be emphasized that C-section rates in both studies were rather low and do not reflect the numbers for the German population in the years the studies were conducted. Thus, it is not possible to make inferences about the prevalence of different interventions based on the study results. However, this was not aim of the present study and would require a research design specializing to this question.

As stated in the method section, Study 1 was part of a broader longitudinal study with different objectives, one of which was measuring the effects of the birth-related mindset on labor and birth outcomes [32]. In the paper[32] it is discussed that using the low-intervention birth index includes some disadvantages, since a rather complex process (labor and birth) is reduced to a dichotomous outcome that weights different interventions equally. However, different measures to calculate interventions (e.g., number of interventions) were tried and the results were all similar to the low-intervention birth index [32]. We therefore argue that the index is an appropriate measure to operationalize the birth process quantitatively.

## Conclusion

The aim of the present paper was to investigate the relevance of partners for the stressful life events of birth, birth experience, and the transition to parenthood. The results suggested that the presence of the partner is positively related to low-intervention birth processes and the birth experience. Furthermore, Study 1 results indicated a positive effect of relationship quality on the transition to parenthood: the higher the relationship quality, the better the well-being during this challenging phase. This effect seems to be independent from gender. Some questions could not yet be answered by the present studies, e.g., questions concerning causality and potential underlying psychological and physiological bases for the found effects. Nevertheless, it became evident that partners serve as an important resource and contribute to psychological well-being during stressful life phases in the medical and family context.

## Data Availability

For both studies data, SPSS syntaxes, and additional results and materials can be found at OSF: https://osf.io/9v2y6/?view_only=23a590304c654489a543535ec021a833.

## Footnotes

1 Since the results do not change when the group *someone else* is not included in the analyses, results are based on the whole sample; see OSF.

2 during data collection, some participants had problems entering the weight and size of the baby. These values were replaced by missing values in the data set. All analyses were performed with a control variable for these cases. No effect on the results could be found; see OSF

3 In an independent data collection (*N* = 2,379) for the years 2020 and 2021 and with only 3% (*n* = 88) without an accompanying person, these results only partially replicated. AUC analyses indicated a slightly higher probability of a low-intervention birth for continuous presence (AUC = 0.53, *p* = .027) but results of logistic regression did not yield significant results for presence or continuous presence. For birth experience results of the initial study could be replicated. Again, low-intervention birth, *B* = 1.184, *SE* = 0.061, *p* < .001, and both presence, *B* = 0.400, *SE* = 0.149, *p* = .007, and continuous presence, *B* = 0.415, *SE* = 0.058, *p* < .001, positively predicted birth experience. See OSF for the detailed results.

4 We also explored relationship quality and continuous midwifery support as potential moderators (also for low-intervention birth as the outcome variable) but results did not yield significant interaction terms. Their results can be found at OSF.
